# Obesity-related pubis lymphedema enclosing genitalia: An atypical case of genital reconstruction

**DOI:** 10.1016/j.eucr.2024.102790

**Published:** 2024-07-02

**Authors:** Daniel-Adrien Wurlod, Joachim Meuli, Anne Favre-Bulle, Nuno Grilo, Pietro G. di Summa

**Affiliations:** a Department of Plastic and Hand Surgery, Centre Hospitalier Universitaire Vaudois (CHUV), University of Lausanne (UNIL), Rue du Bugnon 46, 1011, Lausanne, Switzerland; b Institute of Pathology, Department of Laboratory Medicine and Pathology, Centre Hospitalier Universitaire Vaudois (CHUV), Rue du Bugnon 46, 1011, Lausanne, Switzerland; c Department of Urology, Centre Hospitalier Universitaire Vaudois (CHUV), University of Lausanne (UNIL), Rue du Bugnon 46, 1011, Lausanne, Switzerland

**Keywords:** Massive localized lymphoedema, Obesity, Lymphoedema, Genital reconstruction

## Abstract

Massive localized lymphoedema (MLL) is a rare complication of morbid obesity and has been scarcely reported in the literature, especially in the pubic area and genitalia. It is associated to BMI more than 40 kg/m^2^. We report the case of a 37-year-old patient known for morbid obesity with 68.8 kg/m^2^ BMI and hypogonadism-obesity syndrome presenting an unusually voluminous scrotal MLL mass. Malignancy was ruled out before surgery. In total a 7.5 kg scrotal mass was resected. Surgery was performed with minor complications without requiring additional surgery.

## 1 Introduction

Massive localized lymphedema (MLL) has been scarcely reported in the literature, especially in the pubic area and genitalia, and is associated with morbid obesity and BMI >40 kg/m^2^. Reconstruction in the genital area is challenging as failure can lead to infertility. We report the case of a 37 years old patient known for morbid obesity with 68.8 kg/m^2^ BMI and hypogonadism-obesity syndrome presenting an unusually voluminous scrotal MLL mass.

## 2 Case Presentation

Patient is a 37-year-old male with morbid obesity and BMI of 68.8 kg/m^2^, pre-diabetes, severe obstructive sleep apnea syndrome and hypertension, presenting a growing voluminous scrotal mass over 4 years. Patient's quality of life was severely impacted by his obesity and the scrotal mass, impairing his mobility and spending most of his time in bed. Patient suffered from social withdrawal, explaining the absence of prior investigation.

Initial work-up was made through an abdominopelvic angio-CT scanner showing a voluminous pubic mass enclosing the scrotum of 27 × 35 × 26 cm with adipose density, infiltration of tissue and cutaneous enlargement, along with bilateral inguinal adenomegalies of reactional aspect. Hydrocele or inguinal hernia were ruled out. Needle-biopsies were conducted and showed a fibroadipous and vascular tissue in small abundance with no signs of malignity.

**Fig. 1:**
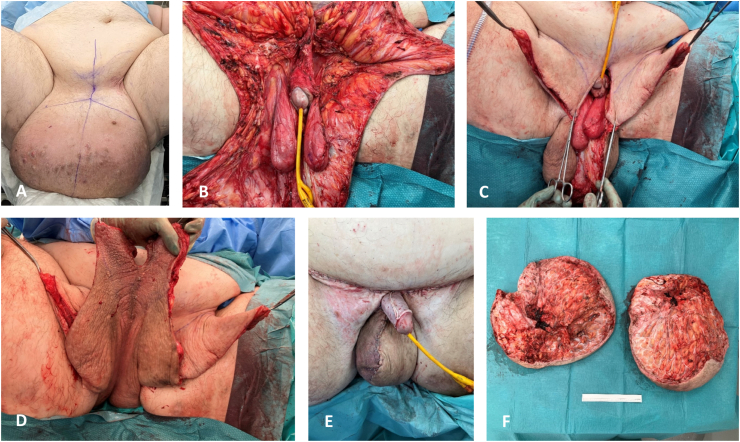
Peroperative surgical sequence: **A.** Preoperative appeareance. **B.** Resection of massive localized lymphedema masses with liberation of gonades and penis. **C.** Proximal excess cutaneous flaps. **D.** Distal excess scrotal cutaneous flap. **E.** Closure using the flaps, a left thigh cutaneous advancement flap for left scrotal reconstruction, and residual cutaneous scrotal flap for right scrotal reconstruction. **F.** Resected masses, 7.5 kg in total.

Surgery was decided, planning for a penile and testicles’ excavation with *in toto* excision of the pubic and scrotal mass. Important lymphatic oozing was observed during dissection. After resection, residual scrotal skin had insufficient vascularization for total scrotal reconstruction. Right half of scrotum was reconstructed with well vascularized scrotal adipocutaneous flap, left half was reconstructed with random freestyle local skin flap of left thigh. Care was taken to keep the available cutaneous perforators included in the flap. Penis shaft was reconstructed with split-thickness skin graft. Mini abdominoplasty was also performed for pubic excess skin and fat resection. In total a 7.5 kg scrotal mass was resected (see Fig. 1).

**Fig. 2:**
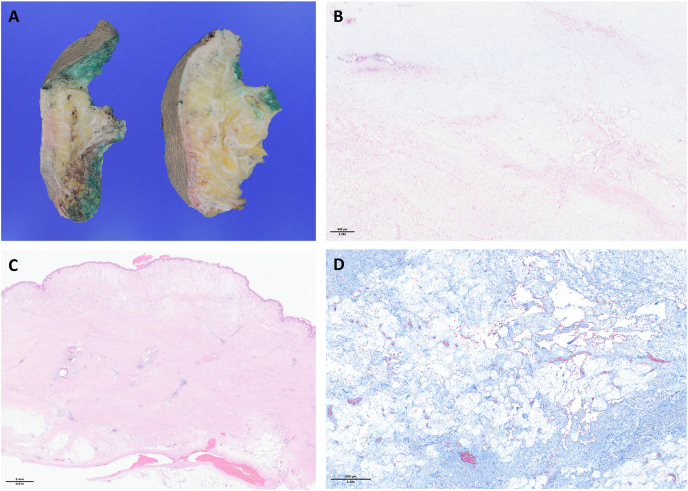
Anatomopathology study: **A. Macroscopy sampling** after formaldehyde fixation. **B. Cutaneous nodule** (hematoxylin and eosin stains): The cutaneous nodules show a thickened collagenous dermis with dermal chronic inflammatory cell infiltrate and loss of skin appendages. **C. Lymphedema** (hematoxylin and eosin stains): Lymphatic proliferation with numerous ectatic lymphatic and plexiform vessels within an edematous paucicellular stroma, without atypia. **D. Transition area** (Masson's Trichrome stains): Masson's Trichrome underlignes the lossely textured collagen within the lymphedema, in contrast to the adjacent thickened dermis, which shows a high collagen density.

Histopathology showed massive and diffuse lymphedema with discrete acute focal inflammation and rare focal liponecrosis, without signs of malignity and without Mdm2 expression, excluding liposarcoma (see Fig. 2).

**Fig. 3:**
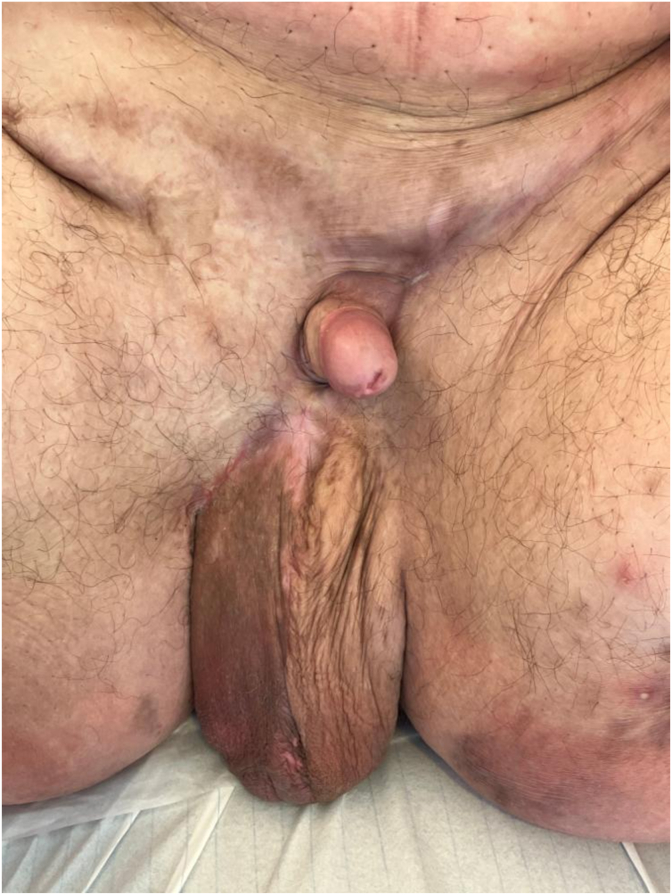
1-year post-operative. **Keywords** Massive Localized Lymphoedema – Obesity – Lymphoedema – Genital reconstruction.

Surgery was followed by minor suture dehiscence in 3 areas (scrotal and inguinal), mainly caused by maceration and lymphatic edema, treated with local wound caring without need for additional surgery. No sign of recurrence is noted 1-year post operatively (see Fig. 3).

## 3 Discussion

Massive localized lymphedema is a pseudotumor characterized by lymphedema chronical aspect and features, such as *peau d’ orange* aspect and skin induration. It is associated to BMI more than 40 kg/m^2^ with average of 60 kg/m2. Legs are predominantly involved but pubic area and genitalia are concerned in 10 % of cases. ^1^ Histologically it shares similarities to well differentiated liposarcoma, thus the importance of excluding malignity especially liposarcoma before planning excision of such lesion. ^2^ Exact pathogenesis remains unknown but main hypothesis is local ischemia due to overweight related compression, stimulating production of growth factor-leading to lobular structure hypertrophy and fibrosis. ^1^^,^^3^^,^^4^ Surgery is the sole treatment but is known for frequent post-operative complications and recurrence rate (up to 50 %). ^5^ Genital reconstruction is challenging, and judging the skin quality and deciding the skin excess usage requires creativity and is of great importance for such cases.

## 4 Conclusion

Massive localized pubic lymphedema enclosing genitalia has been scarcely reported in the literature, with frequent complications and recurrence rate. We report a successful resection and genital reconstruction of such pathology.

## Consent

Patient gave his written consent for usage of picture and clinical data gathered for the present case report.

## CRediT authorship contribution statement

**Daniel-Adrien Wurlod:** Writing – review & editing, Writing – original draft, Validation, Methodology, Formal analysis, Data curation, Conceptualization. **Joachim Meuli:** Validation, Methodology, Investigation, Data curation. **Anne Favre-Bulle:** Validation, Data curation. **Nuno Grilo:** Investigation, Data curation. **Pietro G. di Summa:** Validation, Supervision, Methodology, Investigation, Data curation, Conceptualization.

## Declaration of competing interest

The authors report no conflicts of interest and no conflicts regarding ethics.
